# Efficacy of Enhanced Environmental Cleaning/Disinfection Using Pulsed Xenon Ultraviolet Light in Preventing Outbreaks of Methicillin-Resistant *Staphylococcus aureus* in Neonatal Intensive Care Units

**DOI:** 10.3390/epidemiologia6010012

**Published:** 2025-03-04

**Authors:** Kaori Ishikawa, Toshie Tsuchida, Kaoru Ichiki, Takashi Ueda, Kumiko Yamada, Kosuke Iijima, Naruhito Otani, Kazuhiko Nakajima

**Affiliations:** 1 Department of Infection Control and Prevention, Hyogo Medical University Hospital, Nishinomiya 663-8501, Japan; tsuchida@hyo-med.ac.jp (T.T.); taka76@hyo-med.ac.jp (T.U.);; 2 School of Nursing, Hyogo Medical University, Kobe 650-8530, Japan; 3 Department of Laboratory Technology, Hyogo Medical University Hospital, Nishinomiya 663-8501, Japan

**Keywords:** environmental cleaning/disinfection, methicillin-resistant *Staphylococcus aureus*, neonatal intensive care unit, pulsed xenon ultraviolet light

## Abstract

Background/Objectives: In recent years, non-contact room disinfection devices using ultraviolet light and hydrogen peroxide have emerged as disinfection methods. However, data on their usefulness in neonatal intensive care units (NICUs) are limited. Therefore, the aim of the present study was to evaluate the effectiveness of environmental disinfection in controlling methicillin-resistant *Staphylococcus aureus* (MRSA) outbreaks in a NICU/growing care unit (GCU). Methods: Daily cleaning/disinfection of the patient environment was changed from using a cloth containing quaternary ammonium salts to an agent containing ethanol and surfactant, and terminal cleaning with a pulsed xenon ultraviolet light (PX-UV) non-contact disinfection device was added for patients with confirmed MRSA and those on contact precautions. MRSA incidence and environmental culture results were then compared before and after the method change. Results: The MRSA infection rate was 2.81/1000 patient days before the method change and 0.90/1000 patient days after the change (*p* = 0.008). Environmental cultures were positive in 12/137 (8.8%) before the change and 0 after the change. There were no adverse events in the neonates due to PX-UV irradiation of the environment. Conclusions: Daily cleaning and disinfection with ethanol and surfactant-containing cleaning disinfectants and a final cleaning with a PX-UV non-contact disinfection device reduced environmental MRSA contamination. In addition to adherence to hand hygiene and contact precautions, reducing MRSA present in the environment may contribute to MRSA control in NICUs and GCUs.

## 1. Introduction

Environmental cleaning and disinfection have attracted increasing attention because of the risk of contracting pathogens in contaminated environments. Various pathogens, including vancomycin-resistant enterococci (VRE), *Clostridioides difficile*, *Acinetobacter* spp., and methicillin-resistant *Staphylococcus aureus* (MRSA), can survive on environmental surfaces for several days [1,2], leading to cross-infection when such pathogens adhere to the hands and gloves of healthcare workers [3,4,5]. In addition, patients staying in hospital rooms in which resistant bacteria have been detected are at increased risk of infection from these bacteria [6,7]. Consequently, there is growing interest in environmental cleaning/disinfection as a measure against healthcare-associated infections [1,8,9].

Conventional environmental cleaning/disinfection methods reduce the spread of VRE and MRSA by removing organic matter with cleaning agents using low-concentration (<30%), ethanol-based disinfectants to achieve high disinfection efficacy in a short time [7,9,10,11,12,13,14,15]. However, reports of inadequate cleaning and residual pathogens have been published [16,17]. Non-contact room disinfection methods, such as steam, ultraviolet (UV) light, ozone, and hydrogen peroxide, have recently gained popularity in high-risk environments housing patients with low resistance to infection [18,19,20,21,22]. Non-contact room disinfection equipment can disinfect the intricate shapes and crevices of medical equipment, which is difficult manually. Notably, compared to ozone or other disinfectants that fill the entire room, UV irradiation does not require the sealing of doors and windows or aeration time and is therefore more convenient in a clinical setting, where bed operation must be prioritized. UV radiation is classified as UVC (200–280 nm), UVB (280–320 nm), and UVA (320–400 nm), and its disinfection mechanism involves inducing bacterial deoxyribonucleic acid and viral ribonucleic acid damage [23,24].

Newborns are highly susceptible to acquiring bacteria from healthcare workers and equipment because of their underdeveloped indigenous flora [25,26], which can lead to healthcare-associated infections. *S. aureus* is a problematic pathogen in NICUs worldwide [27], and MRSA, in particular, is difficult to control, because it is a hidden reservoir of infection for many healthcare workers and family members [27,28]. In addition, its ability to survive for long periods of time on environmental surfaces makes it even more difficult to control infection [29]. There were many nosocomial MRSA outbreaks in our NICU/Growth Care Unit (GCU) between 2016 and 2019. In response, referring to the literature, pre-emptive contact precautions were implemented for newborns transported from other hospitals, MRSA carriage was confirmed by the staff, and MRSA-positive patients were cleared of infection [30,31,32]. Furthermore, measures based on epidemiological studies, including the development of nursing procedures, such as timing of hand hygiene and regular skill checks using these procedures, were implemented, resulting in the end of MRSA nosocomial infections [33]. However, another nosocomial outbreak of MRSA in 2020 highlighted the need for further strengthening measures in addition to conventional infection control measures. 

In recent years, the concept of antimicrobial stewardship (AMS) has attracted attention as a core measure to prevent healthcare-associated infections, and the appropriate use of antimicrobials is also recommended in NICUs from the perspective of preventing sepsis [34]. However, MRSA remains one of the major causative agents of healthcare-associated infections not only in Japan but also worldwide, and its control remains an important issue for healthcare institutions in many countries [35,36]. The potential for sepsis, especially in very-low-birthweight infants [37,38,39], calls for the strengthening of comprehensive infection prevention strategies in addition to AMS. Of them, enhanced environmental disinfection may contribute to the control of MRSA transmission in healthcare facilities, but studies of the effectiveness of non-contact room disinfection devices have focused mainly on adult ICUs, with limited reports demonstrating their usefulness in NICUs [40].

This study focused on environmental disinfection as an outbreak control measure and investigated its effectiveness. Environmental disinfection in this study was defined as cleaning and disinfection using agents such as ethanol and surfactants and disinfection using ultraviolet light irradiation equipment. In our hospital, nosocomial infection was defined as a case in which MRSA, which was not detected on surveillance culture tests at the time of admission, was detected within 48 h after admission.

## 2. Materials and Methods

### 2.1. Study Design and Settings

This was a before-and-after study comparing the situation before and after the change in environmental cleaning and disinfection methods. A change was made to the environmental cleaning/disinfection methods of a NICU (15 beds)/GCU (12 beds) at a university hospital in Japan. The NICU and GCU at this hospital were designed with multiple beds arranged in one space rather than in private rooms separated by doors or walls. The period before the change in environmental cleaning/disinfection methods (hereinafter referred to as “before the change”) was 12 months (from 1 July 2020 to 30 June 2021). The period after the change in environmental cleaning/disinfection methods (hereinafter referred to as “after the change”) was also 12 months (from 1 July 2021 to 30 June 2022).

### 2.2. Patient Environment

The patient environment included a ceiling pendant, beds (or incubators), monitoring equipment, a patient-specific wagon, infusion and syringe pumps, ventilators, and other medical equipment used at the patient’s bedside. The ceiling pendant incorporates an electronic medical records system, drainage pipes for oxygen and suction, storage baskets, and an attached workbench.

### 2.3. Before the Change in Cleaning/Disinfection Methods

Nurses were trained to follow and practice environmental cleaning/disinfection procedures, with the wiping order determined by location and frequency of contact. The patient environment was cleaned and disinfected twice daily at 09:00 h and 16:00 h using a cloth containing quaternary ammonium salts (Saracide sterile cloth; Saraya, Chuo-ku, Tokyo, Japan). The cloth frequently dried out during use, preventing the sufficient application of the disinfectant to the target environment.

### 2.4. After the Change in Cleaning/Disinfection Methods

Twice daily, at 09:00 h and 16:00 h, trained nurses applied a cleaning disinfectant containing ethanol and surfactant (Purell Surface; Gojo Japan, Tokyo, Japan) to surfaces in the patient environment and wiped them with gauze (RP Cloth Gauze; Osaki Medical, Aichi, Japan). When MRSA patients and patients under contact precautions were discharged or moved to another patient environment, in addition to cleaning and disinfection with ethanol and surfactant, PX-UV irradiation was performed on the patient environment using a pulsed xenon ultraviolet (PX-UV) device (LightStrike; Xenex Disinfection Services, San Antonio, TX, USA).

### 2.5. PX-UV Irradiation Implementation Method

**Figure 1:**
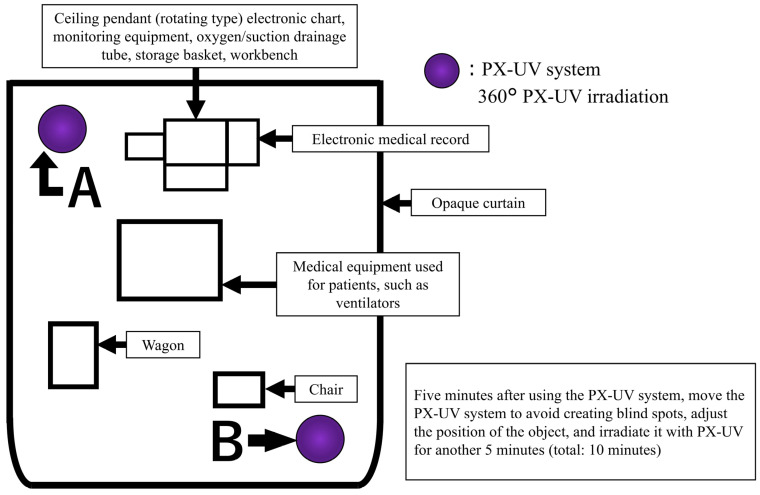
Pulsed xenon ultraviolet (PX-UV) equipment and article placement for the irradiation of patient environments.

The arrangement during PX-UV irradiation is shown in Figure 1. The target patient environment was covered with a light-shielding curtain, and the PX-UV equipment was placed inside the light-shielding curtain. Patients outside the light curtain could not be moved to another room due to therapeutic needs. Patients in beds outside the light curtain had eye protection, and the sensor area was covered with aluminum foil or bedding. PX-UV irradiation was performed from inside the light curtain, and a healthcare professional remained with the patient during irradiation. The PX-UV device was designed to illuminate all spaces in the environment by automatically varying the use of reflectors and the height of the device (2.5′ in the upper position and 2.5′ in the center position). To further reduce shadow areas, unnecessary objects were removed from the patient environment. After irradiating the PX-UV system from position A in Figure 1 for 5 min, the PX-UV system was moved to position B to avoid creating blind spots by adjusting the positions of the medical equipment and articles and irradiated for another 5 min (total 10 min). When it was difficult to adjust the position of the medical equipment or irradiated objects, they were moved to another room, and PX-UV disinfection was performed separately. The incubator used in the NICU was considered too difficult to irradiate without producing shadows; therefore, the patient environment other than the incubator was used as the irradiation target. The infection control staff of the university hospital established procedures for the use of PX-UV equipment and trained NICU/GCU nurses. Training was conducted on the NICU/GCU floor; only nurses who could irradiate the PX-UV device according to the written procedures were responsible for its use. 

### 2.6. Microbiological/Genetic Testing

The environmental culture collection conditions were kept flexible. Specimen collection sites were mostly selected based on patient environments that had been used at least once by one or more MRSA-positive patients. Samples were collected after manual cleaning/disinfection (before change) and PX-UV disinfection (after change). The collection methods involved either directly pressing food stamp standard agar medium (Nissui Pharmaceutical, Tokyo, Japan) onto the target environment or rubbing cotton swabs soaked in saline solution (Kawamoto Sangyo, Osaka, Japan) onto the target environment and storing them in sterile spits (Eiken Chemical, Tokyo, Japan), depending on the area available for collection. Colonies that developed were identified using an automated next-generation microbial identification system (MALDI Biotyper; Bruker Daltonics, Bremen, Germany), and those identified as *S. aureus* were tested for drug susceptibility using the Pos Combo 1J panel (Beckman Coulter, Tokyo, Japan). To confirm the spread of the same strain in the NICU/GCU, the genetic types of the MRSA detected in the environment and in patients were examined. Genetic testing was performed using the polymerase chain reaction (PCR)-based open reading frame typing (POT) method, which determines genotype by detecting 16 open reading frame (ORF) retention patterns in an isolate. This process is called phage ORF typing (POT), a rapid and discriminatory method developed for nosocomial infection control, and it has been reported that MRSA isolates collected in Japan can be genotyped by detecting phage-derived ORF retention patterns with discrimination power comparable to PFGE subtyping, reported to be able to determine the genotype [41], and the PCR-based approach is a practical and efficient alternative for outbreak investigation in clinical settings [41,42].

### 2.7. Data Collected

Birth weight, gestational weeks, number of patients admitted, and duration of hospital stay were collected from electronic medical records, and the monthly hand sanitizer dispensing volume was obtained from medication management information. The calculation method for each is shown below.

1. Number of times hand sanitizer was used per patient per day:
  (amount of hand sanitizer dispensed per month/amount of alcohol hand sanitizer required per visit)/number of patients admitted per month;
2. Hand hygiene compliance rate:
  (World Health Organization’s 5 recommended moments for hand hygiene compliance opportunities/observation opportunities) × 100;
3. Bed occupancy rate:
  (number of hospitalized patients per month/[number of beds × number of days per month]) × 100;
4. MRSA incidence rate (per 1000 patient days):
  (number of MRSA-positive patients/total number of patient days) × 1000.

### 2.8. Statistical Analysis

The obtained values were assessed for power and normality using post hoc testing and the Shapiro–Wilk test, respectively. The chi-squared test was used to compare nominal variables, including patient characteristics, MRSA incidence, and MRSA genetic types, before and after the change. The Mann–Whitney *U* test was used for continuous variables when normality could not be confirmed. The significance level was set at *p* < 0.05. SPSS Statistics for Windows (ver. 24; IBM, Armonk, NY, USA) was used for all statistical analyses.

### 2.9. Ethical Considerations

This study was approved by the Ethics Committee of Hyogo College of Medicine [Reception No. (No. 4351), Management No. (202304-315). and Approval Date: 20 March 2023]. Informed consent was waived, because this was a retrospective, observational study using medical records. All eligible patients’ parents were given the opportunity to refuse participation and opt out via an information disclosure statement. All methods were performed following the relevant guidelines and regulations. The data were accessed for research purposes between 20 March and 31 May 2023.

Since there were no reports on the use of PX-UV in NICUs in Japan, the NICU administrator was asked to implement the following three safety measures, and permission to use PX-UV in this study was obtained.

(1) To ensure the safety of patients in the vicinity of the area subject to environmental cleaning/disinfection, a non-inhalation toxic spray-type disinfectant [43] registered with the US Environmental Protection Agency that had obtained a “Design for Environment” label was used. The International Commission on Non-Ionizing Radiation Protection and the American Council of Governmental Industrial Hygienists have established health hazard standards for continuous irradiation at different UV wavelengths and irradiances [44]. Since the NICU and GCU of this hospital housed multiple patients on a floor with no physical dividing wall, unnecessary areas and patients had to be protected from PX-UV. Therefore, the areas where PX-UV irradiation was necessary were surrounded with light-shielding curtains, and PX-UV was irradiated from inside the light-shielding curtains to ensure that the established health hazard criteria were not met [44]. In addition, healthcare personnel were stationed at the bedside to be ready to respond in case of emergency.
(2) A gap of approximately 10 cm between the light-shielding curtains and the ceiling was created, and there were concerns about the stimulation of nearby patients by flashing lights. Photosensitive epileptic seizures are often induced by flashing light at 10–30 Hz [45,46], but the photosensitive induction range of the PX-UV system is 67 Hz, which does not meet the criteria for inducing photosensitive epilepsy. To be safe, eye masks and towels were used to protect the eyes of nearby patients during irradiation according to the manufacturer’s recommendations (Xenex Disinfection Services).
(3) Despite the PX-UV system’s range of 0–1080 nm, its impact on medical devices (e.g., pulse oximeters) that detect and use infrared radiation has been reported [47]. To prevent exposure of the sensor to infrared radiation, possibly causing abnormal values to be missed, the sensor area of neighboring patients was covered with aluminum foil and bedding. Furthermore, the volume of 60 dB emitted by the PX-UV device did not exceed the American Academy of Pediatrics’ recommended temporary sound pressure or maximum noise level of 65 dB [48], and to prevent persistent noise, using the device for more than 10 min was avoided.

## 3. Results

### 3.1. Comparison of Factors Related to MRSA Outbreak Occurrence

As shown in Table 1, 184 (NICU = 32, GCU = 152) and 194 (NICU = 38, GCU = 156) patients were admitted before and after the change, respectively. No significant differences were noted before and after the change, except for the hand hygiene compliance rate, which improved significantly, from 89.9% before the change to 94.1% after the change (*p* = 0.024).

### 3.2. Use of PX-UV Equipment

The PX-UV system was used 68 times: 30 times in the NICU and 38 times in the GCU. Irradiation protocols were always performed according to the established guidelines. No bed transfers were reported due to any effects during irradiation. In addition, no adverse events, such as epileptic seizures, attributable to PX-UV irradiation were observed in the patients in the NICU/GCU.

### 3.3. Environmental Culture and Detection of MRSA in the Environment and in Patients

The number of samples taken and the number of MRSA detected in the environment are shown in Table 2. Before and after the change in cleaning/disinfection methods, there were 137 sampling locations in nine patient environments (NICU = seven, GCU = two) and 79 sampling locations in five patient environments (NICU = four, GCU = one). In the environmental cultures conducted before the change, 12/137 (8.8%) sampling locations tested positive for MRSA. The 12 locations included (1) patient environments without MRSA-positive patients using beds on the day of environmental culture (eight locations: electronic medical records system keyboard, mouse, bar code reader, storage basket, tray, two-hand windows in incubators, and stethoscopes) and (2) patient environments with MRSA-positive patients using beds on the day of environmental culture (four locations: stethoscope, measuring tape, thermometer, and scissors). After the change, no MRSA was detected at any of the 79 sampling sites in the five patient environments.

The number of MRSA detected in patients and the MRSA incidence rate are shown in Table 3. In the present study, 26 cases of MRSA were detected throughout the entire study period, and before the environmental cleaning/disinfection change, 20 cases (76.9%) were identified, equivalent to 2.81/1000 patient days. After the change, the number of cases decreased significantly to six (23.1%), which corresponds to 0.90/1000 patient days (*p* = 0.008). The power was 0.754.

### 3.4. Susceptibility and Genetic Types of MRSA in Patients and the Environment

Of the *S. aureus* strains detected before and after the change, 40 were identified as MRSA (patient: 26, environment: 14), with cefoxitin ≥8 and oxacillin ≥4 on drug susceptibility testing. The genotypes of MRSA in the environmental culture collection sites and patients are shown in Table 4. Genotype 106-137-80 (hereafter referred to as pattern A) was identified in 10 locations (83.3%) from the environment before the change. Pattern A was also the predominant genetic pattern in patients before and after the change in cleaning and disinfection methods, and it was detected in 11 (55.0%) and 2 (33.3%) cases before and after the change, respectively. This pattern A was presumed to be the same strain.

## 4. Discussion

In the present study, routine cleaning was performed involving agents containing ethanol and surfactants, and PX-UV disinfection was used for the ultimate cleaning step following the established guidelines. Before the change, MRSA with a shared genetic type was identified in both environmental cultures and patients. In contrast, after the change, MRSA was not detected in environmental cultures, and its incidence in patients decreased.

No MRSA was detected in environmental cultures after non-contact terminal cleaning with PX-UV irradiation and the new cleaning/disinfection methods using ethanol and surfactants. Kitagawa et al. [49] reported that PX-UV disinfection and manual cleaning reduced the MRSA acquisition rate, and Morikane et al. [47] reported an 81% reduction in microorganisms with manual cleaning and an additional 59% reduction with PX-UV. These methods can help reduce MRSA contamination in environmental cultures from items that are challenging to clean, such as thermometers, stethoscopes, keyboards, and baskets.

One significant limitation of the present study was the low UV dose in shaded areas, which reduces disinfection efficacy [50]. To address this issue, we developed and implemented guidelines for the placement of items and position of PX-UV irradiation in the NICU/GCU. These efforts, along with training for nurses, led to more effective PX-UV irradiation and improved environmental disinfection. PX-UV disinfection can also be inhibited by organic matter and proteins, and previous studies have shown that MRSA colonies can remain in the environment despite PX-UV disinfection without manual cleaning [51]. To ensure thorough environmental disinfection, it is essential to minimize the presence of PX-UV inhibitors and ensure the quality of routine cleaning through comprehensive training and ongoing monitoring of personnel responsible for cleaning tasks [13,15]. The approach included the systematic manual wiping of areas, with a focus on high-contact areas. Rigorous training sessions were implemented until all nurses were proficient in following the established guidelines. This protocol has been in place since the MRSA outbreak that occurred from 2016 to 2019. A cleaning regimen involving surfactants before PX-UV disinfection and the use of ethanol to eliminate organic matter and proteins, which contributed to the overall effectiveness of PX-UV irradiation, were also implemented.

The cleaning and disinfection method transitioned from using quaternary ammonium salts to using a solution containing ethanol and surfactants to ensure an adequate contact time with disinfectants for effective disinfection, which can be challenging in busy clinical settings. It has been reported that quaternary ammonium salts are not expected to be effective in quaternary ammonium salt formulations if they are used to the extent that they dry after a contact time shorter than 1 min [52]. In addition, cloths containing quaternary ammonium salts often dry out during use, which may result in inadequate disinfection. To ensure effective disinfection, our new method involves spraying a necessary amount of disinfectant onto pathogen-contaminated surfaces and wiping with gauze, which has shown efficacy even with a short contact time of approximately 30 s [43].

In the present study, since outbreak prevention measures were continuously implemented, hand hygiene compliance remained consistently high (over 80% before and after the intervention). The study period coincided with the global outbreak of Coronavirus Disease 2019 (COVID-19), during which hand hygiene was reinforced as an infection prevention measure. As a result, hand hygiene compliance improved not only in the NICU but also throughout the entire hospital.

It is crucial for healthcare workers to maintain proper hand hygiene to prevent the spread of MRSA [53,54]. A study by Song et al. [55] demonstrated that, in the NICU, when hand hygiene compliance was below 80%, the risk of MRSA acquisition increased, and even with additional MRSA countermeasures, sufficient effectiveness could not be achieved. In the present study, by maintaining a hand hygiene compliance rate of over 80% and implementing comprehensive MRSA countermeasures, the MRSA acquisition rate was reduced by 48%.

In addition to the high hand hygiene compliance rate in this study, improvements in environmental cleaning and disinfection practices may have contributed to the reduction in MRSA acquisition rates. Furthermore, the heightened awareness of infection prevention measures due to the COVID-19 pandemic may have led to behavioral changes by healthcare workers, which could have affected the high hand hygiene compliance rate and the decrease in MRSA infection rates.

## 5. Limitations

This study had several limitations. First, because it was validated in the context of an MRSA outbreak response, it did not meet the requirements of an experimental study, limiting the comparability of environmental cleaning/disinfection methods. In addition, the ambient environment of all MRSA-detected patients was not sampled, and samples were not collected at the end of wiping with the modified cleaning/disinfectant. Moreover, samples were not taken at the end of wiping with cleaning disinfectant after the change. In addition, the contamination status of other patient environments was unknown, because the environmental culture collection sites were limited to patients in whom MRSA had been detected. Second, this study did not examine comparability by medical procedures or patients’ physical characteristics as confounding conditions. Finally, the present study was performed in a single hospital under outbreak conditions. Interventional studies in controlled settings are needed for generalizability.

Despite these limitations, the results of the present study suggest that enhanced environmental cleaning and disinfection methods may be effective for preventing MRSA outbreaks and that UV disinfection can be implemented safely in NICUs/GCUs.

## 6. Conclusions

Daily cleaning and disinfection with ethanol and surfactant-containing agents, along with non-contact terminal cleaning using PX-UV, resulted in no MRSA detection in environmental cultures and a decrease in the incidence of MRSA in the NICU/GCU. Furthermore, the improved compliance rate of hand hygiene also contributed to the decrease in MRSA incidence. In addition to practicing proper hand hygiene, strengthening environmental cleaning and disinfection methods may help prevent the spread of MRSA in the NICU/GCU.

## Figures and Tables

**Table 1:** Comparison of the factors affecting MRSA outbreak occurrence.

Factor Affecting MRSA ^a^ Outbreak Occurrence		Cleaning/Disinfection Method		*p*-Value
		Before Change *n* = 184	After Change *n* = 194	
Mean birth weight (g)		2259 ± 701.2 (342–3972)	2387 ± 776.7 (621–4542)	0.104
Average gestational weeks		36 ± 3.4 (27–41)	36 ± 3.6 (24–41)	0.337
Bed occupancy rate (%)	NICU ^b^	80.1	76.6	0.512
	GCU ^c^	66.7	61.0	0.261
Average duration of hospital stay (days)	NICU	17.6 ± 27.2 (1–113)	10.6 ± 21.2 (1–100)	0.172
	GCU	32.4 ± 31.3 (2–201)	29.6 ± 29.1 (2–185)	0.230
No. of hand sanitizations per patient per day (times)		97.8	91.2	0.343
Hand hygiene compliance rate (%)		89.9	94.1	0.024

Data are expressed as the mean ± standard deviation (min–max) values. ^a^ MRSA: methicillin-resistant *Staphylococcus aureus*, ^b^ NICU: neonatal intensive care unit, and ^c^ GCU: growing care unit.

**Table 2:** Number of samples collected, and MRSA detected in the environment.

Sample Collection Conditions	Cleaning/Disinfection Methods					
	Before Change			After Change		
	Patient Environments (*n*)	No. of Collected Samples (Location)	No. of Detected MRSA ^a^ Locations (Locations)	Patient Environments (*n*)	No. of Collected Samples (Location)	No. of Detected MRSA ^a^ Locations (Locations)
Patient environment used at least once by a patient with MRSA detected	9	137	12	2	28	0
Patient environment with MRSA detected in environmental culture prior to cleaning/disinfection change	–			3	51	0

^a^ MRSA: methicillin-resistant *Staphylococcus aureus*.

**Table 3:** Number of MRSA detected in patients and incidence of MRSA.

	Cleaning/Disinfection Methods		*p*-Value
	Before Change	After Change	
Number of MRSA detected in patients	20	6	–
Incidence of MRSA/1000 patient days	2.81	0.90	0.008

**Table 4:** Environmental culture collection locations, numbers of samples, and genetic types of MRSA at the NICU/GCU before and after the change in cleaning/disinfection methods.

		Genetic Type * of MRSA in Patients		Environmental Cultures Number of Samples/MRSA Counts (Genetic Type *)	
Bed Number		Before	After	Before	After
NICU	1				
	2	A	I	16/0	
	3	B			
	4	A	G		18/0
	5	B		18/4 (A,A,A,A)	
	6	A		17/3 (A,A,A)	31/0
	7				
	8	A			
	9	E			
	10	B		17/0	
	11	A			
	12	A,B,B		16/3 (A,A,A)	10/0
	13	D,E	A,H,A	11/2 (I,I)	10/0
	14	A,A		16/0	
	15				
GCU	1				
	2				
	3				
	4				
	5	A	F	16/0	10/0
	6				
	7				
	8				
	9				
	10	A			
	11	A		10/0	
	12	C			

* A: 106-137-80, B: 93-80-9, C: 93-13-51, D: 95-64-9, E: 95-80-9, F: 70-18-81, G: 106-217-35, H: 106-183-40, and I: 106-183-37.

## Data Availability

The original contributions presented in this study are included in the article.
